# Gradient Engineered Light Absorption Layer for Enhanced Carrier Separation Efficiency in Perovskite Solar Cells

**DOI:** 10.1186/s11671-020-03359-0

**Published:** 2020-06-09

**Authors:** Gaozhu Wu, Qing Zhu, Teng Zhang, Ziqi Zou, Weiping Wang, Yiyan Cao, Lijing Kong, Xuanli Zheng, Yaping Wu, Xu Li, Zhiming Wu, Junyong Kang

**Affiliations:** Department of Physics, Fujian Key Laboratory of Semiconductor Materials and Applications, Collaborative Innovation Center for Optoelectronic Semiconductors and Efficient Devices, Xiamen University, Xiamen, 361005 People’s Republic of China

**Keywords:** Perovskite solar cells, Gradient band structure, Carrier separation efficiency, Carrier recombination loss

## Abstract

Carrier transport behavior in the perovskite light absorption layer significantly impacts the performance of perovskite solar cells (PSCs). In this work, reduced carrier recombination losses were achieved by the design of a band structure in perovskite materials. An ultrathin (PbI_2_/PbBr_2_)_n_ film with a gradient thickness ratio was deposited as the lead halide precursor layer by a thermal evaporation method, and PSCs with a gradient band structure in the perovskite absorption layer were fabricated by a two-step method in ambient atmosphere. For comparison, PSCs with homogeneous perovskite materials of MAPbI_3_ and MAPbI_*x*_Br_3 − *x*_ were fabricated as well. It is found that the gradient type-II band structure greatly reduces the carrier lifetime and enhances the carrier separation efficiency. As a result, the PSCs with a gradient band structure exhibit an average power conversion efficiency of 17.5%, which is 1–2% higher than that of traditional PSCs. This work provides a novel method for developing high-efficiency PSCs.

## Introduction

In the last 10 years, perovskite solar cells (PSCs) have become the focus of attention in the field of energy because of their high efficiency and low cost [1–6]. Many efforts have been focused on the improvement of cell performance or power conversion efficiency (PCE) [7–13]. As we know, the cell performance essentially depends on incident photon-to-electron conversion efficiency (IPCE) and light absorption efficiency, whereas efficient carrier separation efficiency is the key to improve IPCE. Therefore, it becomes crucial to control the transport of photon-generated carriers in perovskite materials. In traditional planar structure PSCs, carriers (or electron-hole pairs) are separated at the interface between the electron transfer layer (ETL) and perovskite material, and only carriers diffusing to the separation interface can have an effect on cell performance. Hence, much work has been devoted to reduce carrier recombination during its diffusion. Different methods, such as solvent annealing [14–17], additive engineering [18–20], surface passivation [21–24], etc. have been employed to improve perovskite crystal quality. Actually, band alignment control is an alternative method to reduce recombination losses [25–27]. For example, Jing Zhang et al. introduced extrinsic movable ions Li^+^/I^−^in MAPbI_3_, and the aggregation of Li^+^/I^−^ tuned the energy level of the perovskite, which made charge extraction quite efficient from perovskite materials to both ETL and hole transport layer (HTL) in PSCs [28]. Interestingly, the band structure of organometal halide perovskite materials can be easily adjusted by changing the component or content of the halogen element in perovskite materials [29–32]. For instance, Zhang et al. fabricated a MAPbI_3_/MAPbI_*x*_Br_3 − *x*_ heterostructure with a type-II band structure and achieved HTL-free PSCs [33]. In essence, it is an ideal approach to directly reduce recombination losses through the design of a gradient band structure in the perovskite light absorption layer [34, 35], which supports the carrier separation as fast as possible. However, to the best of our knowledge, it has not been reported so far about the fabrication of perovskite materials with a gradient band structure.

In this work, ultrathin PbI_2_ and PbBr_2_ films were alternately deposited onto the substrate as the lead halide precursor layers by a thermal evaporation method, and PSCs with a gradient engineered perovskite absorption layer were fabricated by gradually tuning the thickness ratio of PbI_2_ to PbBr_2_ films. For comparison, PSCs with homogeneous perovskite materials of MAPbI_3_ and MAPbI_*x*_Br_3 − *x*_ were fabricated as well. Scanning electron microscopy (SEM), energy dispersive X-Ray spectroscopy (EDS), X-ray diffraction (XRD), absorption spectra, photoluminescence (PL) spectra, and time-resolved photoluminescence (TRPL) spectra were performed to investigate the morphologies, element distribution, crystal structures, chemical compositions, optical properties, and carrier lifetime of perovskite materials. It is found that the gradient band structure in the perovskite light absorption layer significantly reduces the carrier lifetime and enhances the carrier separation efficiency. As a result, the PSCs with a gradient band structure exhibit an average power conversion efficiency of 17.5%, which is 1–2% higher than that of traditional PSCs.

## Experimental Section

### Device Fabrication

**Fig. 1:**
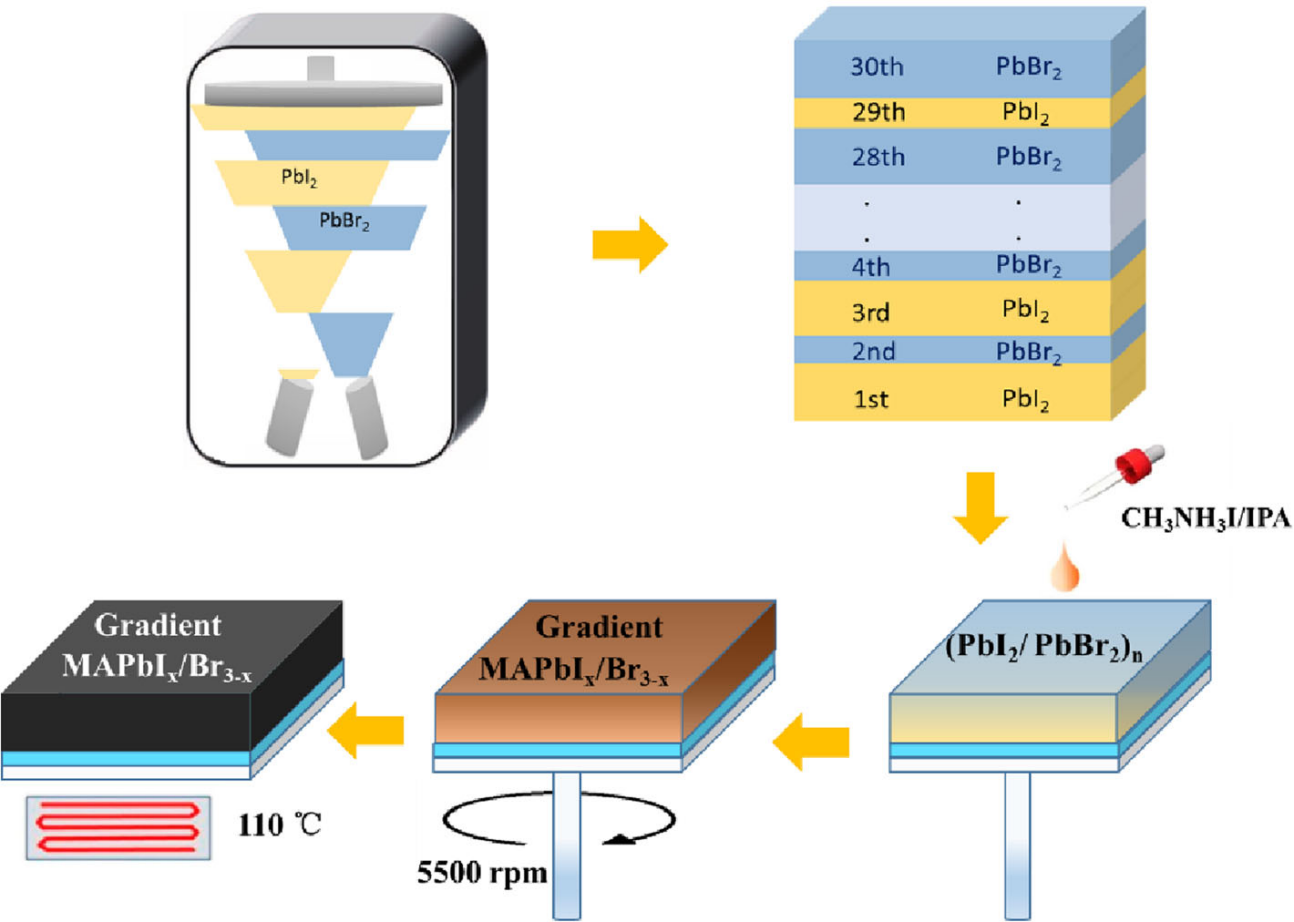
Schematics of the fabrication process of gradient MAPbI_*x*_Br_3 − *x*_ perovskite films

Fluorine-doped tin oxide (FTO) glass substrates (15 Ω/sq) were etched by a laser and cleaned by sequential ultra-sonication in acetone, ethanol, and deionized water for 15 min in each. A compact SnO_2_ (c-SnO_2_) layer was deposited on the cleaned FTO substrates by spin-coating 0.1 M tin oxide ethanol solution (Xi’an Polymer) at 3000 rpm for 30 s, and then annealed at 200 °C for 120 min. After the substrates were naturally cooled to room temperature, they were immersed in tin tetrachloride solution for 20 min at 75 °C, and then rinsed with deionized water and dried by nitrogen flow. Three kinds of perovskite layers, i.e., MAPbI_3_, MAPbI_*x*_Br_3 – *x*_, and G-MAPbI_*x*_Br _− *x*_, were prepared by a two-step method. To fabricate homogeneous MAPbI_3_ or MAPbI_*x*_Br_3 − *x*_ perovskite films, PbI_2_ (99.99%, Xi’an Polymer) with a thickness of 180 nm was first evaporated on the prepared substrate at a rate of 0.5 nm/s. To convert lead halides to MAPbI_3_ materials, the precursor film with a solution of CH_3_NH_3_I (MAI) in isopropanol (40 mg/mL) was spin-coated on the substrates, whereas to convert lead halides to MAPbI_*x*_Br_3 − *x*_, a MAI and MABr (CH_3_NH_3_Br) mixed isopropanol solution (mole ratio: 4:1) was used as the precursor and spin-coated on the as-prepared FTO/c-SnO_2_/PbI_2_ substrates. As for the fabrication of gradient MAPbI_*x*_Br_3 − *x*_ perovskite films, PbI_2_ and PbBr_2_ were alternately evaporated onto the FTO/c-SnO_2_ substrate as the lead halide precursor layers, as shown in Fig. 1, by accurately controlling the evaporation time of PbI_2_ and PbBr_2_, and a 180-nm heterogeneous lead halide layer with a gradient thickness ratio, consisting of (11.6 nm PbI_2_/0.4 nm PbBr_2_)/(11.2 nm PbI_2_/0.8 nm PbBr_2_)/....../(6 nm PbI_2_/6 nm PbBr_2_), was obtained. And then the precursor film with a solution of MAI in isopropanol (40 mg/mL) was spin-coated on the as-prepared FTO/c-SnO_2_/(PbI_2_/PbBr_2_)_15_ substrates. All the above spin-coating speeds were set to 5500 rpm, and all the samples were annealed at 110 °C for 60 min. The hole transport material (HTM) was deposited by spin-coating at 4000 rpm for 30 s, which was composed of 72 mg of spiro-OMeTAD, 28.8 μL of 4tert-butylpyridine, and 17.5 μL of 520 mg/mL lithium bis-(triflouromethanesulfonyl) imide in acetonitrile in 1 mL of chlorobenzene. All the above processes are operated in air atmosphere with a relative humidity of 35%. Finally, 80-nm-thick gold electrodes were deposited on the top of the devices by a thermal evaporation method. The active area of the cell was measured to be 0.07 cm^2^.

### Characterization

Morphologies of the samples and EDS mapping images were examined by a high-resolution field emission SEM (FE-SEM, Zeiss Sigma). Crystal structures were analyzed by XRD (Ultima IV, Rigaku, Cu Kα: *λ* = 0.15406 nm). The absorption spectra of perovskite films were characterized by an UV/Vis spectrophotometer (PerkinElmer, Lambda 850). The current density-voltage (J-V) curves were measured by a digital source meter (B2901A, Keysight) under an AM 1.5 solar simulator (SS150, Zolix). The IPCE was measured in AC mode on a (QE-R, Spectral Response Measurement System) testing system (Enli Technology Co. Ltd.) with a tungsten-halogen lamp as the light source. PL and TRPL spectra were measured by a steady-state transient near-infrared fluorescence spectrometer (FLS 980) at a laser wavelength of 377 nm as an excitation source.

## Results and Discussion

**Fig. 2:**
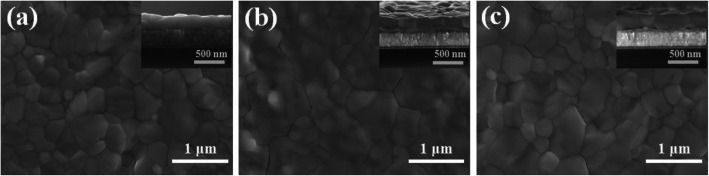
The surface and cross-sectional morphologies of perovskite films: **a** MAPbI_3_, **b** MAPbI_*x*_Br_3 − *x*_, and **c** G-MAPbI_*x*_Br_3 − *x*_. The inserts are their cross-sectional images

The surface and cross-sectional morphologies of the perovskite films were characterized by SEM. Figure 2 a–c show the surface images of the perovskite samples of MAPbI_3_, MAPbI_*x*_Br_3 – *x*_, and gradient MAPbI_*x*_Br_3 − *x*_ (labeled as G-MAPbI_*x*_Br_3 − *x*_), respectively. The inserts are their cross-sectional images. All the samples exhibit a uniform and compact surface, indicating the good crystal quality of perovskite materials. Notably, the samples demonstrate the different grain sizes. The MAPbI_*x*_Br_3 − *x*_ sample has an average grain size in the order of micrometers, whereas for the MAPbI_3_ and G-MAPbI_*x*_Br_3 − *x*_ samples, the grain sizes are ~ 350 nm and ~ 450 nm, respectively. The size difference should be related to the material growth process. As for the MAPbI_*x*_Br_3 − *x*_ sample, a MAI and MABr mixed isopropanol solution was used as the precursor, whereas, for the other two samples, only the MAI isopropanol solution was adopted. Br atoms in the precursor solution tend to slowly displace I atoms during the growth process because of their different atomic radius, which is conducive to reduce the growth rate and increase the grain size. This behavior is also observed in other reports [36, 37]. In addition, as shown in the inserts of Fig. 2, the thickness for all three samples is controlled to ~ 350 nm, which is similar to the optimized value in the previous report [38].

**Fig. 3:**
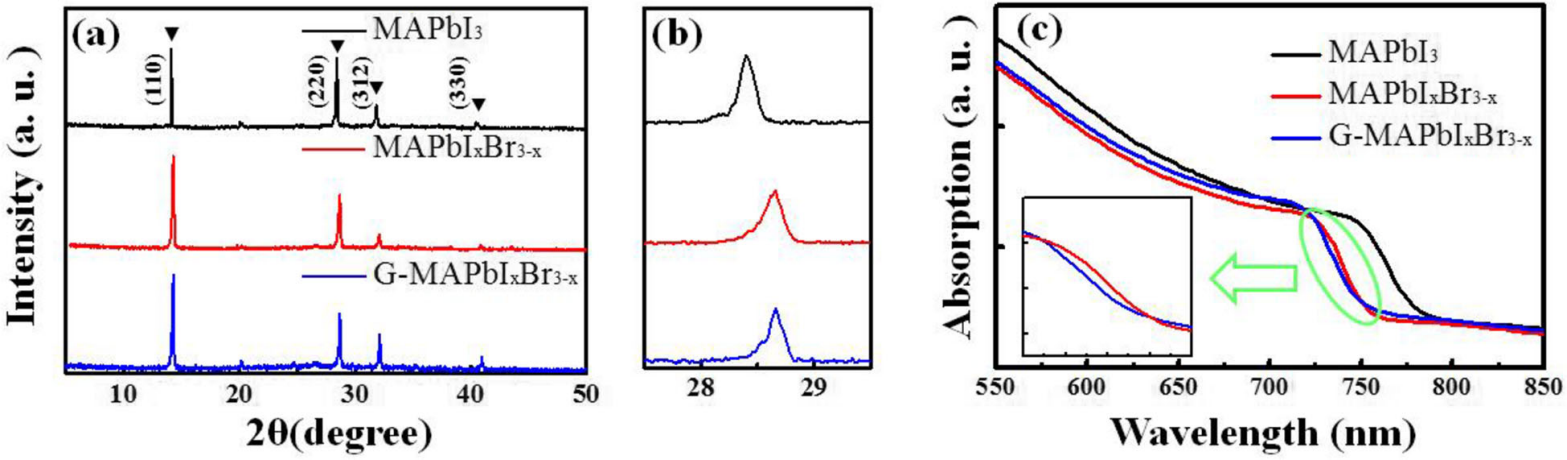
**a** XRD patterns of perovskite films, **b** their partial enlargement, and **c** absorption spectra

To elucidate the crystal structures and compositions of different samples, XRD measurements were performed with the results in Fig. 3a. The diffraction peaks at around 14.1°, 28.4°, 31.8°, and 40.9° are indexed to (110), (220), (312), and (330) planes of MAPbI_3_ material, respectively. There is no characteristic peak corresponding to hexagonal PbI_2_ or PbBr_2_, revealing the complete conversion. With respect to the MAPbI_3_ sample, as shown in Fig. 3b, the diffraction peaks of the MAPbI_*x*_Br_3 − *x*_ and G-MAPbI_*x*_Br_3 − *x*_ samples slightly shift to the larger 2*θ* degrees and almost appear in the same position, indicating the analogous doping content of Br atoms [32]. Figure 3 c shows their absorption spectra. For the MAPbI_3_ sample, an obvious absorption edge at ~ 785 nm is observed, corresponding to a bandgap of 1.58 eV. For the other two samples, both the absorption edges move to ~ 755 nm, corresponding to a bandgap of 1.64 eV. Empirically, the bandgap *E*_*g*_ can be estimated by the following quadratic equation:

$$ {E}_g\left( MAPb{I}_x{Br}_{3-x}\right)=2.29-0.35x+0.037{x}^2, $$

hence the Br content can be estimated to be ~ 16% [32]. It is worth noting that although the MAPbI_*x*_Br_3 − *x*_ and G-MAPbI_*x*_Br_3 − *x*_ samples have almost the same absorption edges, their curve slopes exhibit a certain difference. The absorption edge for the G-MAPbI_*x*_Br_3 − *x*_ sample is relatively gentle, which might be because it is a non-homogeneous perovskite material possessing a non-identical bandgap.

**Fig. 4:**
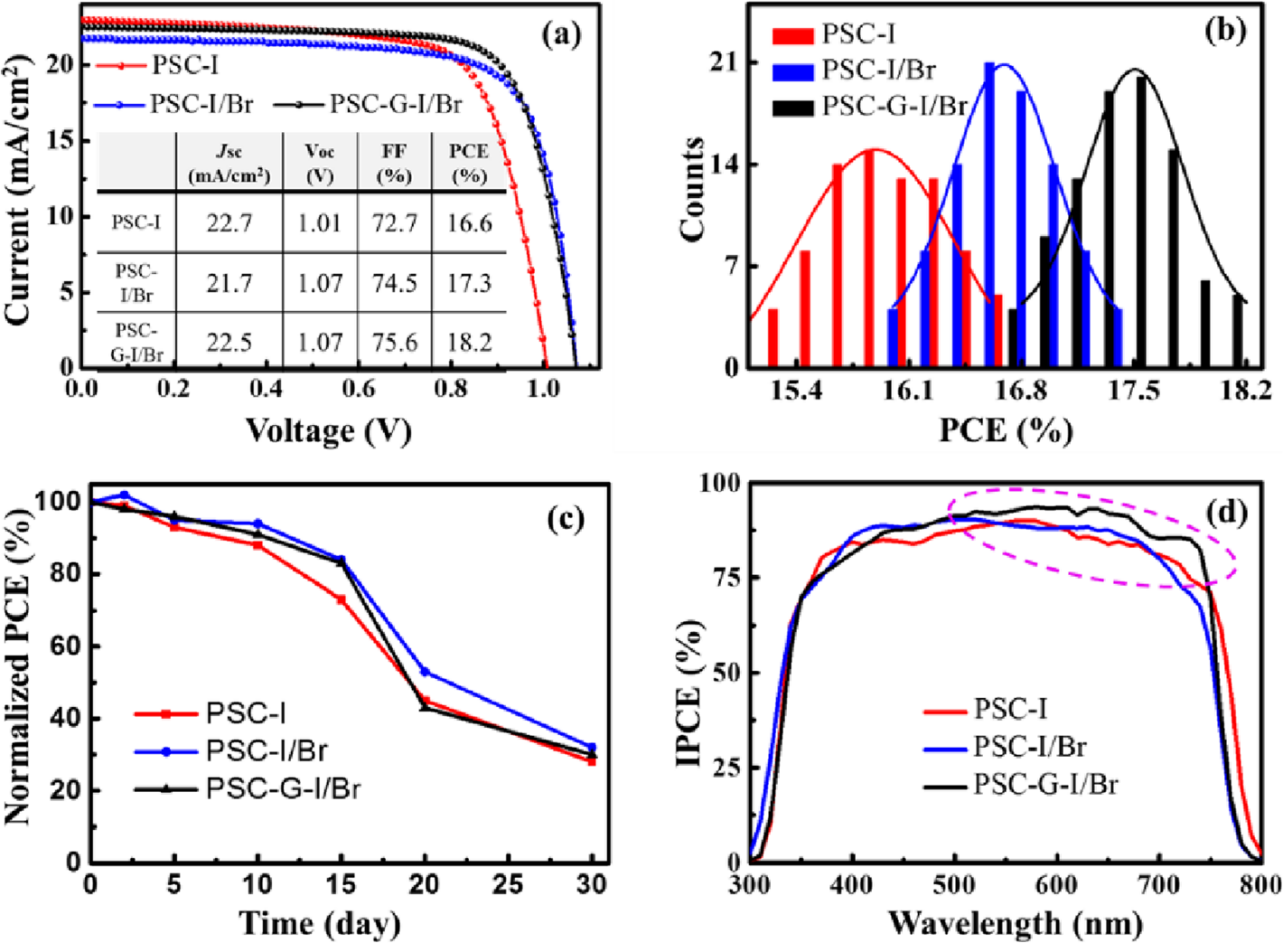
**a** J-V curves of the PSCs, the insert is their parameter results. **b** The PCE histograms of PSC devices. **c** PCE evolution of unencapsulated devices under dark storage in a dry box (25 °C, RH 30%). **d** IPCE curves of the PSCs

The PSCs were fabricated by using the above three samples and labeled as PSC-I, PSC-I/Br, and PSC-G-I/Br, respectively. Figure 4 a displays the J-V characteristic curves. The detailed performance parameters are summarized in the inserted table. It can be seen that the PSC-G-I/Br exhibits the best performance with a PCE of 18.2%, corresponding to an open-circuit voltage (Voc) of 1.07 V, a short-circuit current density (Jsc) of 22.5 mA/cm^2^, and a filling factor (FF) of 75.6%. Understandably, the Voc of both the Br-containing cells is 0.06 V higher than that of the cell without the Br component, since the Br-doping expands the bandgap of the perovskite material and improves the Voc [1]. In addition, compared with the other two cells, the PSC-I/Br cell has a significantly reduced Jsc (21.7 mA/cm^2^). This might be because of the less light absorption in the perovskite material owing to its larger bandgap. To confirm the effectiveness of the experiment, we fabricated 80 devices for each kind of cell. Figure 4 b exhibits the PCE histograms. Obviously, the PSC-G-I/Br devices have the highest average PCE of 17.5%, whereas the PSC-I and PSC-I/Br devices exhibit the lower average PCEs, corresponding to ~ 15.8 % and ~ 16.7%, respectively. Figure 4 c shows the stability results. After three weeks, the cell performance is almost reduced by 60%. Here, it should be mentioned that our experiment is fully operated in air atmosphere (relative humidity 35%), and the PSCs with higher efficiency and stability can be hopefully achieved when they are fabricated in a low-humidity environment.

To reveal the mechanism of the improved performance in PSC-G-I/Br devices, the measurements of IPCE, PL, EDS, and TRPL for the different samples were performed. Figure 4 d shows their IPCE curves with the wavelength range from 300 to 800 nm. There are slight differences in cutoff wavelength and IPCE intensity. As for the PSC-I device, it shows the largest effective region from 300 to 780 nm, contributing to the maximal Jsc. By contrast, the Br-containing devices (i.e., PSC-I/Br and PSC-G-I/Br) exhibit the shorter cutoff wavelength due to the larger bandgap. Notably, compared with the PSC-I/Br device, the PSC-G-I/Br device has the higher IPCE in the range from 500 to 750 nm, resulting in the larger Jsc. This phenomenon may be related to the light absorption distribution in the perovskite material. It is known that the extinction coefficient of the perovskite material decreases with the increase of light wavelength in the visible range [39]. Therefore, when sunlight is incident on the perovskite cell, the short-wavelength light tends to be absorbed in the region close to the separation interface owing to its small penetration depth, and the photo-generated carriers have the higher separation efficiency, while the long-wavelength light has the deeper penetration depth, and more photo-generated carriers locate far away from the separation interface, which is not conducive to carrier separation. Accordingly, as shown in Fig. 4d, for the PSC-I/Br or PSC-I device, the IPCE in the long wavelength is slightly lower than that in the short wavelength. However, for the PSC-G-Br/I device, the gradient energy band structure contributes more to the improvement of the carrier separation efficiency far away from the interface than near the interface. Hence, a significant improvement of IPCE appears in the long wavelength (500–750 nm).

**Fig. 5:**
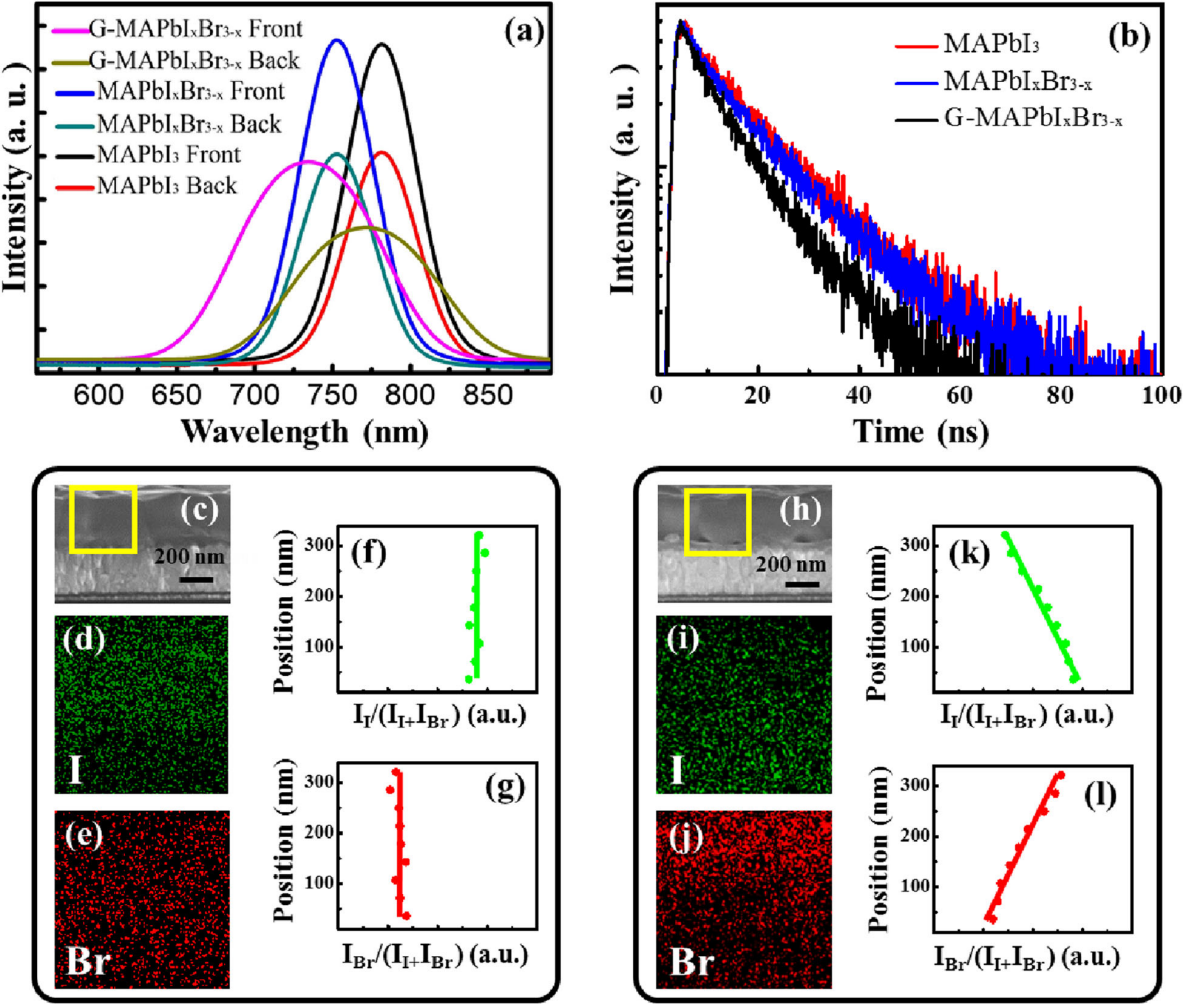
**a** Steady-state PL spectra. **b** TRPL spectra. **c** Cross-section image of an MAPbI_*x*_Br_3 − *x*_ film. **d**, **e** EDS mapping images of I and Br elements in the area marked in (**c**), respectively. **f**, **g** Relative intensity of I and Br element based on (**d**) and (**e**) images along the longitudinal direction. **h** Cross-sectional image of a G-MAPbI_*x*_Br_3 − *x*_ film. **i**, **j** EDS mapping images of I and Br elements in the area marked in (**h**), respectively. **k**, **l** Relative intensity of I and Br element based on (**i**) and (**j**) images along the longitudinal direction

Figure 5 a shows their PL spectra measured from the front (from perovskite material) and the back (from the glass). In the case of MAPbI_3_ and MAPbI_*x*_Br_3 − *x*_ materials, the PL peak positions locate at 780 and 752 nm, respectively, corresponding to the bandgap of 1.58 and 1.64 eV, which agree well with the results in Fig. 3c. Meanwhile, the peak positions of PL spectra measured from the different sides are the same, demonstrating the homogenous materials. Interestingly, for the MAPbI_*x*_Br_3 − *x*_, the two PL spectra exhibit the different peak positions locating at 734 nm and 771 nm, respectively, corresponding to a bandgap difference of 80 meV; moreover, the full width at half maximum (FWHM) of PL spectra is broader than that of MAPbI_3_ or MAPbI_*x*_Br_3 − *x*_ materials. These phenomena should be related to the non-homogeneous Br distribution in perovskite materials. As for the G-MAPbI_*x*_Br_3 − *x*_ sample, it is equivalent to multi-component material, and the PL spectrum is composed of multiple spectra, resulting in the broadening of FWHM. In addition, when excitation light is incident on different sides, each single spectrum contributes differently to the total PL spectrum. In the case of the PL spectrum measured from the front, more contributions are from the surface perovskite material with the larger bandgap, resulting in the peak position of the PL spectrum locating at the shorter wavelength. And vice versa, the peak position of the PL spectrum measured from the back locates at the longer wavelength. To further analyze the Br element distribution, EDS mapping of I and Br elements were performed for the MAPbI_*x*_Br_3 − *x*_ and G-MAPbI_*x*_Br_3 − *x*_ samples. As shown in Fig. 5c–g, the I and Br elements almost uniformly distribute on the whole perovskite layer for the MAPbI_*x*_Br_3 − *x*_ sample, whereas a gradient I and Br distribution along the longitudinal direction can be clearly observed for the G-MAPbI_*x*_Br_3 − *x*_ as shown in Fig. 5h–l, and moreover, the closer to the FTO substrate, the smaller the Br content. These results are consistent with the original expectation. In addition, it can be seen from Fig. 5a that the PL intensity for the G-MAPbI_*x*_Br_3 − *x*_ material is markedly lower than that for the other two samples. As we know, the emission intensity is significantly impacted by the carrier lifetime of perovskite material. Figure 5 b plots the TRPL spectra measured at 770 nm for different samples. We fitted the carrier lifetime through a two-component exponential decay function [40]:

$$ F(t)=A+{B}_1\exp \left(\frac{-t}{\tau_1}\right)+{B}_2\exp \left(\frac{-t}{\tau_2}\right), $$

where *A* is the baseline offset constant, *B*_1_ and *B*_2_are the corresponding attenuation amplitudes of this component, and *τ*_1_ and *τ*_2_ are the decay time. The average recombination lifetime (*τ*_ave_) can be calculated by the following equation:

$$ {\tau}_{ave}=\frac{\sum {B}_i{\tau}_i^2}{\sum {B}_i{\tau}_i}. $$

The fitting values of *τ*_*ave*_ for MAPbI_3_ and MAPbI_*x*_Br_3 − *x*_ and G-MAPbI_*x*_Br_3 − *x*_, are 18.4 ns, 18.1 ns, and 13.1 ns, respectively. It can be seen that the G-MAPbI_*x*_Br_3 − *x*_ sample has the shortest carrier lifetime. As we know, material quality can also impact the carrier lifetime, and a poor quality will result in a short carrier lifetime [41–43]. According to our XRD results shown in Fig. 3a, the characteristic peaks at 14.1° are sharp and their FWHMs are almost same for the three samples, which demonstrate that there is little difference in their crystal qualities [15, 43]. In addition, no broadening appears for the FWHM of PL spectrum in MAPbI_*x*_Br_3 − *x*_ compared with that in MAPbI_3_, as shown in Fig. 5a, demonstrating that our fabrication technology is suitable for the preparation of perovskite materials with mixed halogen elements. Furthermore, in our experiment, the PSC-G-MAPbI_*x*_Br_3 − *x*_ exhibits the higher PCE compared with another two kinds of cells, which goes against the poor quality of the G-MAPbI_*x*_Br_3 − *x*_ material. Hence, it is reasonable to believe that the shorter decay time in TRPL spectrum is mainly attributed to the gradient band structure and the higher carrier separation efficiency in the G-MAPbI_*x*_Br_3 − *x*_ material. In this sense, the designed gradient band structure in our experiment is beneficial for the carrier separation and device performance compared with traditional homogeneous band structure.

**Fig. 6:**
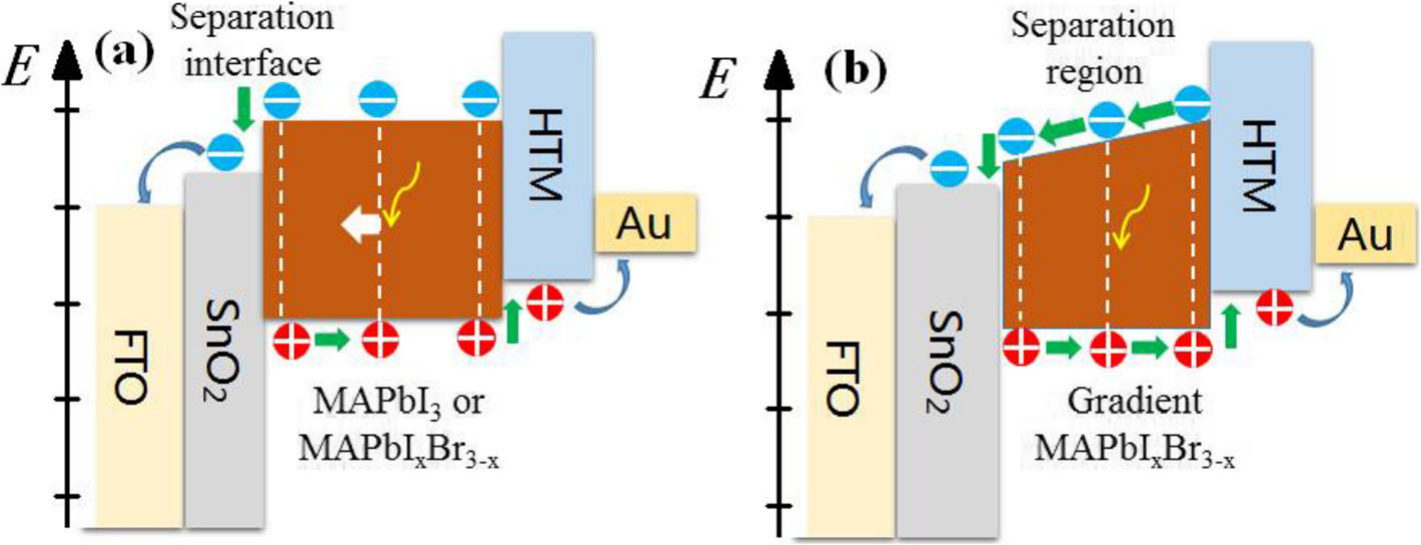
Schematic diagrams of the working principle for different PSCs. **a** PSC without a gradient type-II band structure. **b** PSC with a gradient band structure

In order to further elaborate how the gradient band structure affects the performance of PSCs, schematic diagrams of the working principle for PSCs with or without a gradient band structure in the perovskite material were drawn in Fig. 6. As for the traditional structure PSC shown in Fig. 6a, electron-hole pairs are firstly generated in perovskite materials under light irradiation, and then they are separated at the interface between SnO_2_ and perovskite material, leading to the current output. From this point of view, only the electron-hole pairs diffusing to the separation interface can contribute to the output current. Hence, improving crystal quality has been extensively employed to enhance cell performance due to the increased number of electron-hole pairs reaching the separation interface. Based on previous reports [44], the conductive band for lightly Br-doped perovskite material (MAPbI_*x*_Br_3 − *x*_) will gradually increase with the Br content while the valance band almost keeping fixed. In the light of this, the band structure for the PSC with a gradient Br content is drawn in Fig. 6b. Compared with the traditional band structure shown in Fig. 6a, the gradient structure supports carrier separation inside perovskite materials, which significantly reduces carrier radiative or non-radiative recombination during the diffusion process, thereby improving the carrier separation efficiency and cell performance.

## Conclusions

In this work, perovskite absorption layers with a gradient or non-gradient band structure were designed and fabricated by a two-step method, and three kinds of PSCs, i.e. PSC-I, PSC-I/Br and PSC-G-I/Br, were achieved. The results reveal that the gradient band structure in perovskite absorption layers is beneficial for the reduction of carrier recombination losses. An enhanced carrier separation efficiency and IPCE was achieved in the PSC with a gradient band structure. As a result, the kind of PSCs exhibits an average PCE of 17.5%, which is 1–2% higher than that of traditional PSCs. This work paves a way to design high-efficiency PSCs.

## Data Availability

We declared that materials described in the manuscript, including all relevant raw data, will be freely available to any scientist wishing to use them for non-commercial purposes, without breaching participant confidentiality.
